# Efferent Loop Syndrome in a Post-pancreatoduodenectomy Patient Due to Exuberant Foreign Body Giant Cell Reaction Mimicking Cancer: A Case Report

**DOI:** 10.7759/cureus.4904

**Published:** 2019-06-14

**Authors:** Humaid Ahmad, Omema Saleem, Muhammad Zeeshan Raza, Jahanzaib Haider, Shams Nadeem Alam

**Affiliations:** 1 Surgery, Dow University of Health Sciences, Karachi, PAK; 2 Surgery - Breast Surgery, Civil Hospital Karachi, Dow University of Health Sciences, Karachi, PAK; 3 Surgery, Civil Hospital Karachi, Dow University of Health Sciences, Karachi, PAK

**Keywords:** efferent loop syndrome, whipple's pancreatoduodenectomy, foreign body giant cell reactions, completion extended cholecystectomy, gallbladder carcinoma, extended cholecystectomy

## Abstract

Efferent loop syndrome has been rarely reported after pancreatoduodenectomy. In those cases that have been reported, the majority presented late and recurrence or peritoneal metastases were found to be the usual causes. Foreign body giant cell reactions (FBGCR) also rarely develop into masses that are large enough to cause problems or mimic malignancy. This report presents a case of a middle-aged female who underwent completion extended cholecystectomy for carcinoma of the gallbladder. Whipple’s pancreatoduodenectomy was also performed at the same surgery due to presence of a hard mass at the cystic duct stump that was densely adherent to the common bile duct and duodenal cap. This was later found to be FBGCR. The patient underwent re-exploration just three weeks later for efferent loop syndrome, the cause of which was again found to be a mass due to FBGCR that was not previously present. Despite a difficult initial treatment phase, the patient is disease free and doing well after two and half years of completing treatment for the carcinoma gallbladder.

## Introduction

“Loop Syndromes” include efferent loop syndrome (ELS) and afferent loop syndrome (ALS). They are purely mechanical complications encountered following certain varieties of gastric surgery where gastrojejunostomy is constructed. The mechanical problem that occurs in ELS and ALS is the obstruction of the outflow of either efferent or afferent limb, respectively. These two syndromes may be difficult to differentiate due to similar symptoms [1]. Although ALS is more commonly reported in literature, very little data has been published regarding ELS making it a much rarer entity [1, 2]. In ELS, obstruction of the efferent limb of the gastrojejunostomy results in gastric outlet obstruction [3]. When ELS occurs early after surgery, poor surgical technique is usually the cause [1, 2]. Intestinal hernias, bands, adhesions and recurrence of malignancy lead to delayed presentation [1-3]. With regard to pancreatoduodenectomy, ELS usually occurs late with the main cause being recurrence of malignancy [3]. In this case report, a case of a middle-aged female is reported who underwent completion extended cholecystectomy (CEC) and Whipple’s pancreatoduodenectomy (WPD) for carcinoma gallbladder and presented with the rare ELS in the early postoperative period. The cause of the ELS was found to be a fibrous mass of tissue due to foreign body giant cell reaction (FBGCR). Despite successful management and even disease-free survival after two and half years of completing treatment for carcinoma gallbladder, exuberant FBGCR seemed to have played a disproportionately unwarranted role in the patient’s initial management.

## Case presentation

A 38-year-old otherwise healthy female presented with a two-year history of right hypochondrial pain consistent with the diagnosis of biliary colic. Her laboratory workup (including liver function tests) was normal. An ultrasound of her abdomen revealed multiple gallstones and a 1 cm x 1 cm single polyp in the body of her gallbladder. She underwent open cholecystectomy with an unremarkable postoperative outcome. The histopathology report of her gallbladder revealed a 1.2 cm x 1.5 cm lesion in the wall of her gallbladder consistent with Grade II (moderately differentiated) adenocarcinoma. It was a T1b lesion with uninvolved cystic duct margin. The case was discussed in the tumor board meeting of our hospital and it was recommended that CEC should be undertaken followed by gemcitabine-based adjuvant chemotherapy.

CEC was planned and the patient was operated three weeks after the first surgery. Peroperatively, the patient was found to have a 1 cm x 1 cm hard mass at the cystic duct stump, which was a new finding. This mass was densely adherent to the common bile duct (CBD) and the first part of the duodenum. Because malignancy was suspected and the mass could not be freed from the duodenum, combined WPD and CEC was undertaken. The patient had an uneventful recovery and was discharged on her tenth postoperative day. The histopathology report of her second surgery revealed no evidence of malignancy and the hard mass was found to be due to FBGCR. However, no foreign body was identified in the specimen. Review of the operative notes of the first surgery revealed that the cystic duct had been ligated using silk suture.

**Figure 1:**
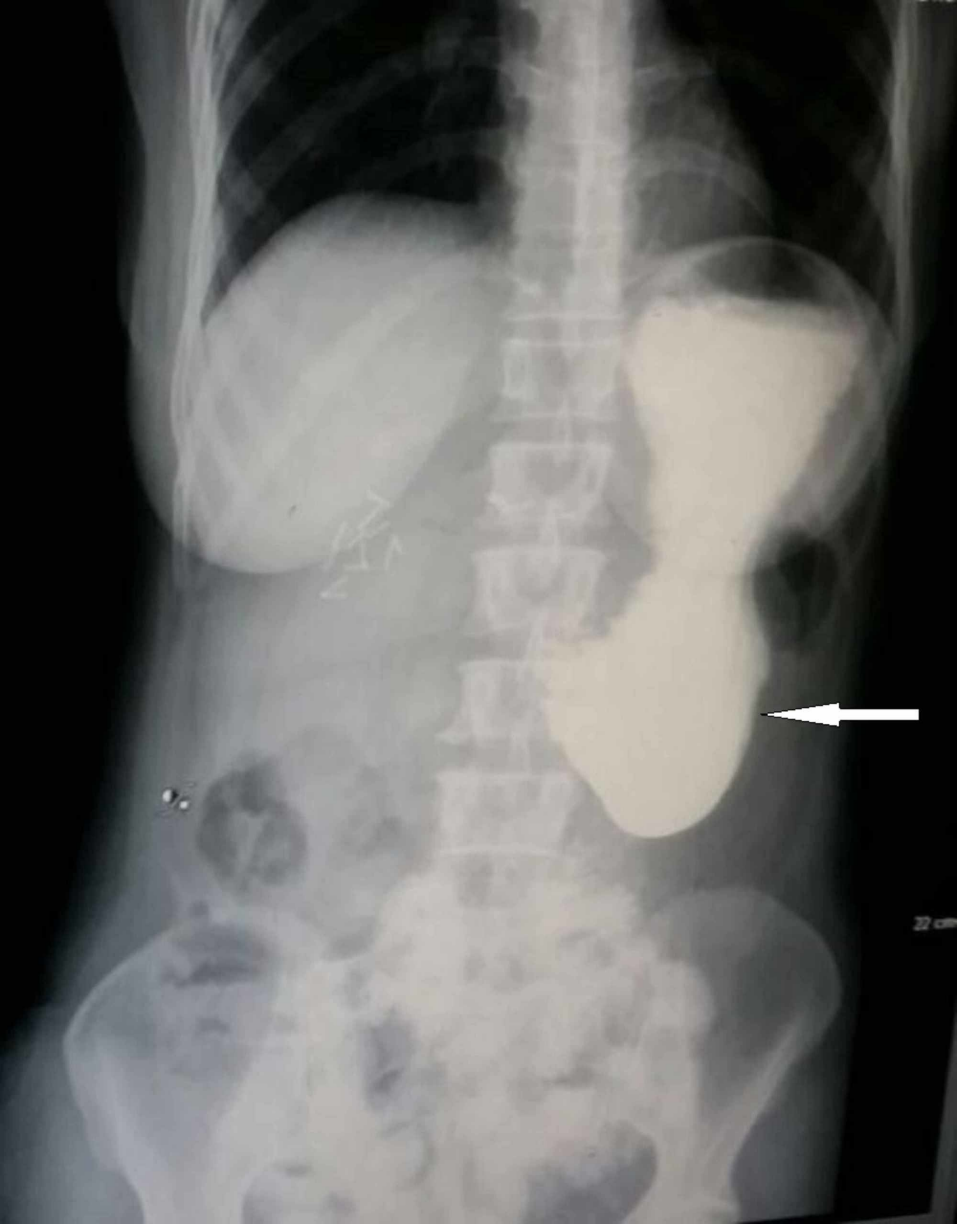
Barium meal of patient post completion extended cholecystectomy and Whipple's pancreatoduodenectomy. White arrow shows hold up of dye in the dilated stomach.

The patient presented on her fourteenth postoperative day with complaint of on and off non-bilious vomiting for two days. The usual upper GI disturbances that occur after WPD were suspected and she was treated with dietary modification, pro-kinetic and anti-emetic agents. However, she had no relief in her symptoms. The symptoms increased in intensity and became persistent by the end of the third postoperative week. She did not develop jaundice during this period at any time. The patient was re-admitted for further evaluation. Her laboratory data was unremarkable including liver function tests. A diluted barium meal study was advised that showed hold up of barium in a dilated stomach with minimal dye passing into the efferent limb of the Roux-en-Y gastrojejunostomy (Figure 1). Upper GI endoscopy was also performed and the gastrojejunostomy site was passed using the scope. While insufflation of air dilated the afferent limb and bile was visualized flowing in the expected direction, insufflation did not result in dilatation of the efferent limb. Additionally, the scope could not be negotiated further than 2 to 3 cm into the efferent limb where a sudden kinking was encountered.

**Figure 2:**
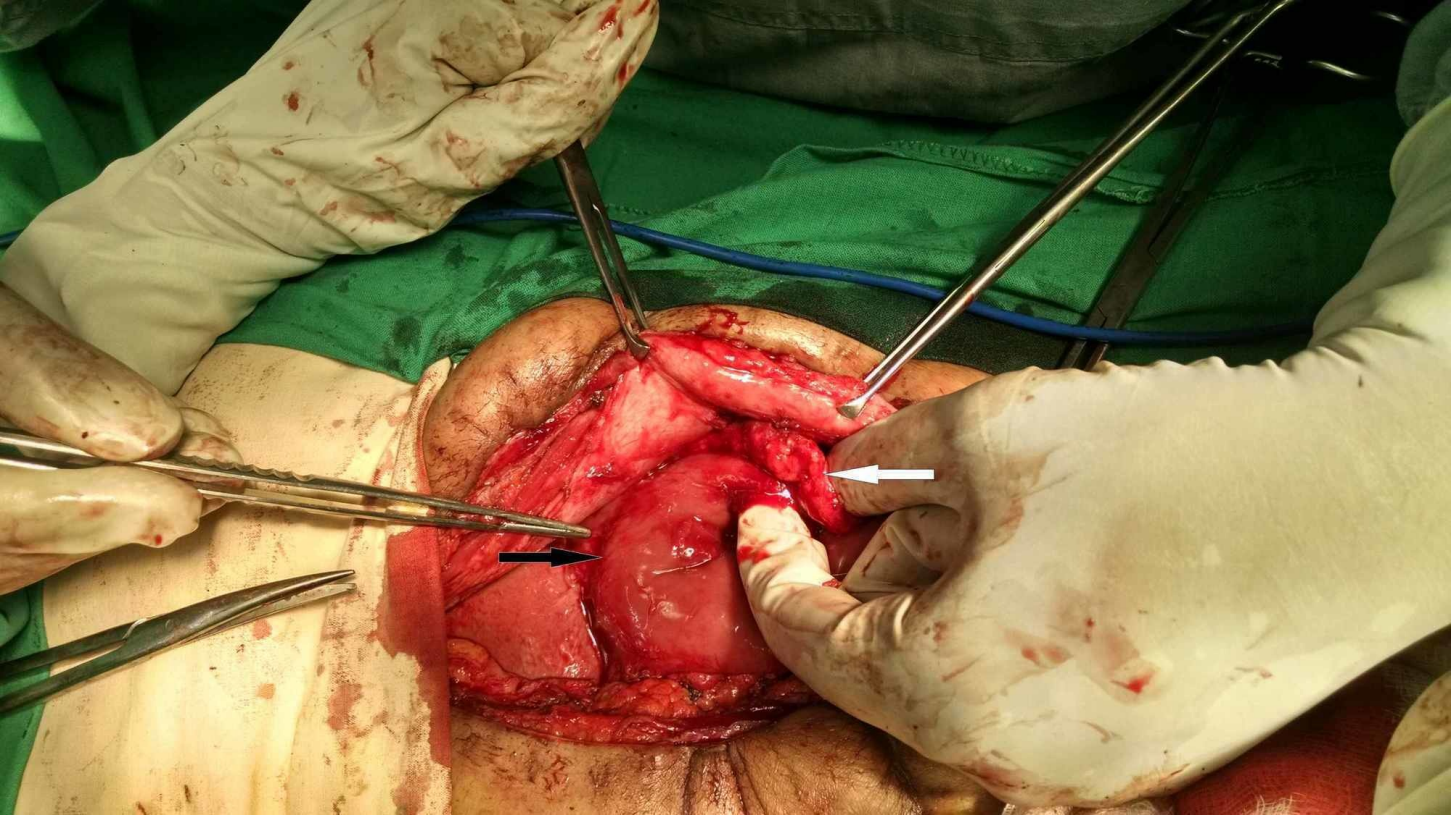
Per-operative picture shows proximity of hard mass to the gastrojejunostomy site. White arrow shows the hard mass between the anterior abdominal wall and the efferent limb of the Roux-en-Y reconstruction. Black arrow shows the gastrojejunostomy site.

**Figure 3:**
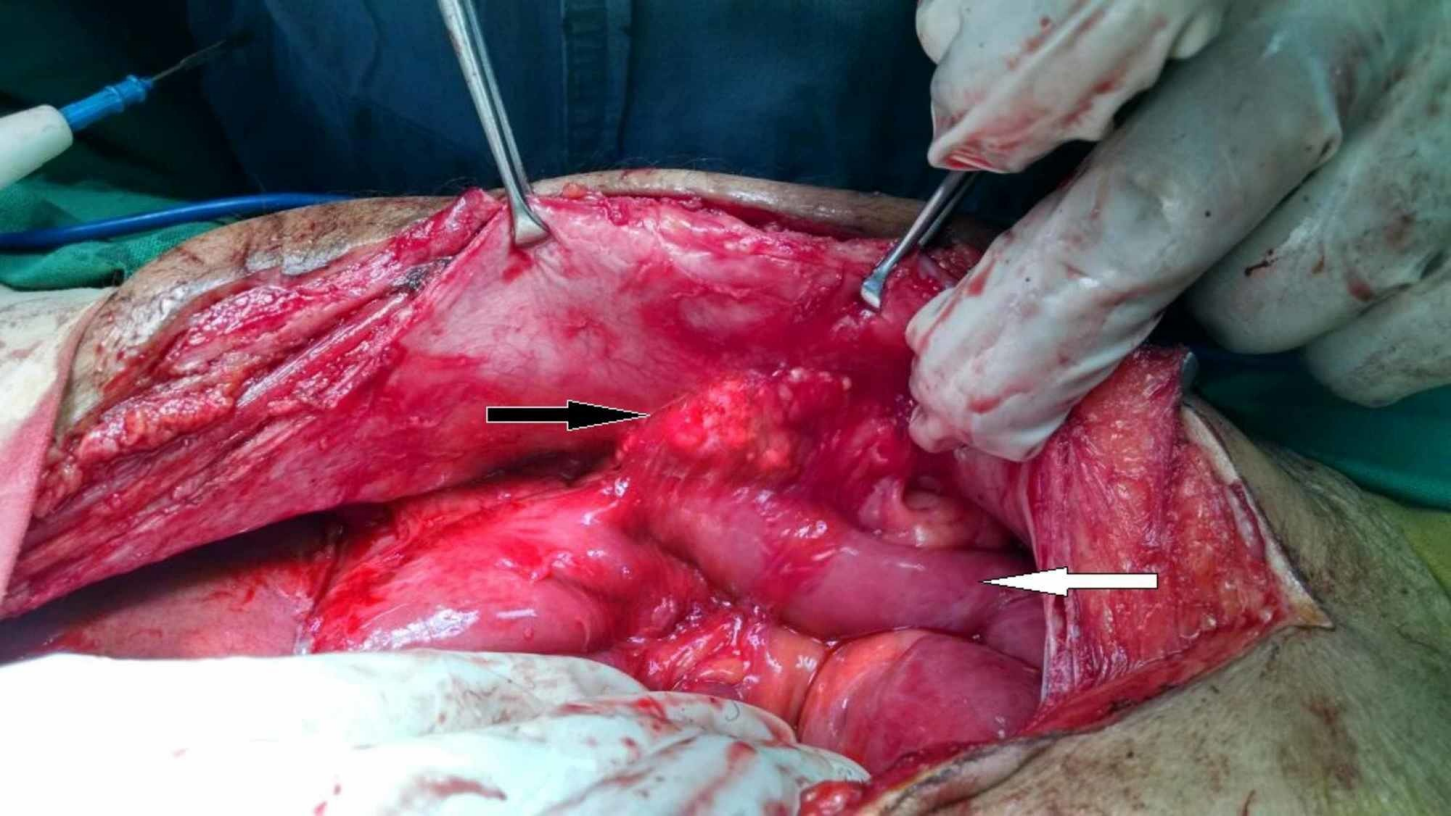
Per-operative picture showing hard mass adhered between anterior abdominal wall and efferent limb of Roux-en-Y gastrojejunostomy. Black arrow shows hard mass between anterior abdominal wall and efferent limb of Roux-en-Y reconstruction. White arrow shows efferent limb of Roux-en-Y reconstruction.

On the basis of the above investigations, the patient was suspected to have efferent loop syndrome and was planned for urgent surgery. Peroperatively, an approximately 3 cm x 3 cm hard mass was found that was densely adherent to the inner side of the laparotomy incision and to the efferent limb of the gastrojejunostomy approximately 2 to 3 cm from the gastrojejunostomy site. There was significant kinking of the efferent limb because of this mass (Figures 2, 3). Apart from some flimsy adhesions around the gastrojejunostomy site, the rest of the peritoneal cavity revealed no abnormalities.

This mass was dissected free from the small bowel and abdominal wall. As the previous histopathology report had revealed FBGCR and only three weeks had passed since her curative surgery, it was suspected that this may also be a mass of similar origin rather than peritoneal metastasis. Since the laparotomy incision had been previously closed with polypropylene (Prolene®) suture, it was decided to close the incision with polyglactin-910 (Vicyl®) suture this time. The patient had an unremarkable recovery and was discharged on her tenth postoperative day after she was tolerating oral soft diet. The histopathology report of the resected mass again showed FBGCR. The patient had no further symptoms and she was referred for adjuvant chemotherapy. Two and a half years after her adjuvant chemotherapy for gallbladder cancer, the patient is disease free and doing well.

## Discussion

ELS is a rare syndrome where the patient suffers high intestinal obstruction [4]. It is related to the efferent limb of the gastrojejunostomy that is created as an alternate route for drainage of stomach contents. A common cause of ELS in the early postoperative period has been found to be poor surgical technique [1]. While ELS has been associated with gastric surgical procedures [1], an extensive literature search revealed it has been rarely reported after pancreatoduodenectomy. Cases of ELS that were found after pancreatoduodenectomy were mainly due to recurrence of malignancy and occurred late after the initial surgery [3]. In this case, the patient was only in the third week of her postoperative period when her symptoms began. As the problem is related to the outflow limb of the gastrojejunostomy, the typical symptoms of ELS are copious bilious vomiting, abdominal discomfort relieved by vomiting, and upper abdominal distention where symptoms may be worst at night [2,4]. However, if a Braun jejunojejunostomy (an anastomosis between the afferent and efferent limb of the gastrojejunostomy) has been constructed during the initial procedure and the site of efferent loop obstruction is proximal to this anastomosis, bile juice may not be a substantial part of the vomitus. This is because this anastomosis allows bile and pancreatic secretions to drain directly into the distal jejunum with minimal passage through stomach [2,5]. As we had constructed a Braun jejunojejunostomy in this patient during the initial surgery, this explains the relatively non-bilious vomiting in our patient.

Barium meal using water-soluble contrast can be expedited quite easily and will demonstrate holdup of contrast in a massively dilated stomach (Figure 1). This should be the investigation of choice in the early postoperative period in such patients [2]. There may be passage of small amount of contrast into the jejunum as occurred in our patient, but one must remember that there should be free flow of contrast into the jejunum from the stomach without a dilated stomach showing holdup of contrast. Upper GI endoscopy can be of great benefit in reaching the diagnosis by showing sudden kinking of the efferent limb and being unable to negotiate the scope further down. However, it does have a risk of breakdown of anastomosis [2]. A combination of the above two investigations can be used to rule out anastomotic site edema and delayed gastric emptying as the cause of patient’s symptoms, which are conditions that can be managed conservatively [2] and also occur in the early postoperative period. CT scan may be ordered to confirm these findings. However, a CT scan was not done after the barium meal and upper GI endoscopy in this case as we suspected some error in surgical technique to be the cause of the problem. The early clinical presentation and absence of clinical features suggesting metastasis or disease progression led us to this conclusion. As such, an urgent laparotomy was planned as the next step in management.

It is interesting to see that FBGCR was the cause of development of hard masses on two occasions in this patient. FBGCR are elicited by all implantable biomaterials. Tissue macrophages, due to their ability to recognize self and non-self, attach to foreign materials in an attempt to ingest and degrade them. A failed attempt at ingestion leads to fusion of adjacent macrophages to produce multinucleated giant cells, representing FBGCR. Through a complex set of pathways under control of various cytokines, a process of chronic inflammation ensues. This leads to fibrous encapsulation of the implanted materials. This intense inflammatory process can even lead to exuberant tissue fibrosis and scarring during the remodeling phase of wound healing [6]. While physical features of implanted materials such as size, substrate stiffness and topography seem to determine the extent of FBGCR [6], non-biodegradable property seems to be most important feature with regard to sutures. This explains the intense FBGCR that occurred after the first and second surgery in our patient. Although histopathology report did not reveal suture material in the specimen, the presence of large masses at the sites where silk and polypropylene (both non-absorbable sutures) were used leads us to this conclusion.

As pancreatoduodenectomy is a major undertaking fraught with major complications, we feel it is unfortunate that our patient underwent this procedure because of a mass that was later discovered to be due to FBGCR. However, FBGCR has been reported to be the cause of formation of masses that were mistaken for malignant deposits [7-10]. These masses have even led to surgical resection of the suspected areas [8,10]. As frozen section was not available in the hospital, one of two affiliated hospitals where our hepatopancreatobiliary team worked at the time of treatment of this patient, it was deemed that the major procedure was the only way forward for the patient at that time. This is not only because of the high index of suspicion of malignancy, but also because experienced surgeons were available and the procedure could be undertaken safely. What is left to be discussed is the fact that, on two occasions, our patient suffered a very intense FBGCR with development of hard masses within a period of just three weeks. What patient factors were involved in exhibition of this tendency to develop such intense reaction over a very short period of time remains unclear and cannot be explained by just one case.

## Conclusions

ELS is rare and can occur after pancreatoduodenectomy even in the early postoperative period. It should be suspected if the patient presents with intractable and persistent vomiting in the early postoperative period especially if symptoms are not responding to conservative management. Contrast studies and upper GI endoscopy suffice to make the diagnosis in early postoperative period. Exuberant FBGCR may produce large masses in the early postoperative period that can lead to this syndrome. Any newly formed intraabdominal masses found during completion surgery or re-exploration in the early postoperative period of cancer surgery should be viewed with caution when planning further surgical treatment as they may not be representative of malignant deposits. Availability of frozen section facility can aid in avoiding misinterpretation.
